# Gillespie eco‐evolutionary models (GEMs) reveal the role of heritable trait variation in eco‐evolutionary dynamics

**DOI:** 10.1002/ece3.1959

**Published:** 2016-01-18

**Authors:** John P. DeLong, Jean P. Gibert

**Affiliations:** ^1^ School of Biological SciencesUniversity of Nebraska – LincolnLincolnNebraska68588

**Keywords:** Body size, consumer–resource dynamics, Eco‐evolutionary dynamics, ecological pleiotropy, Gillespie algorithm, trait variation

## Abstract

Heritable trait variation is a central and necessary ingredient of evolution. Trait variation also directly affects ecological processes, generating a clear link between evolutionary and ecological dynamics. Despite the changes in variation that occur through selection, drift, mutation, and recombination, current eco‐evolutionary models usually fail to track how variation changes through time. Moreover, eco‐evolutionary models assume fitness functions for each trait and each ecological context, which often do not have empirical validation. We introduce a new type of model, Gillespie eco‐evolutionary models (GEMs), that resolves these concerns by tracking distributions of traits through time as eco‐evolutionary dynamics progress. This is done by allowing change to be driven by the direct fitness consequences of model parameters within the context of the underlying ecological model, without having to assume a particular fitness function. GEMs work by adding a trait distribution component to the standard Gillespie algorithm – an approach that models stochastic systems in nature that are typically approximated through ordinary differential equations. We illustrate GEMs with the Rosenzweig–MacArthur consumer–resource model. We show not only how heritable trait variation fuels trait evolution and influences eco‐evolutionary dynamics, but also how the erosion of variation through time may hinder eco‐evolutionary dynamics in the long run. GEMs can be developed for any parameter in any ordinary differential equation model and, furthermore, can enable modeling of multiple interacting traits at the same time. We expect GEMs will open the door to a new direction in eco‐evolutionary and evolutionary modeling by removing long‐standing modeling barriers, simplifying the link between traits, fitness, and dynamics, and expanding eco‐evolutionary treatment of a greater diversity of ecological interactions. These factors make GEMs much more than a modeling advance, but an important conceptual advance that bridges ecology and evolution through the central concept of heritable trait variation.

## Introduction

The effect of ecological processes on evolutionary dynamics has long been acknowledged, but ecologists have historically dismissed the possibility of evolution affecting ecological dynamics in the short term based on the assumption that evolutionary processes occur on longer timescales than ecological ones (Thompson 1998, 2005; Hairston et al. 2005). Both experiments and theory, however, increasingly show that evolutionary and ecological processes can occur on similar timescales (Hairston et al. 2005; Palkovacs and Hendry 2010; Schoener 2011; DeLong et al. 2016). Although it is now clear that ecological changes are generally faster than evolutionary changes (DeLong et al. 2016), trait changes are often fast enough to cause feedbacks or downstream effects on ecological dynamics. Examples of evolutionary trait change directly affecting population dynamics cover a wide range of systems from short‐lived predator–prey systems of rotifers and algae (Yoshida et al. 2003) to long‐lived ungulates evolving in response to changing environmental conditions (Ozgul et al. 2009). These studies suggest that it is critical to develop modeling approaches to characterize and predict the consequences of eco‐evolutionary dynamics.

A key determinant of how fast evolution occurs and whether it has the potential to interact with short‐term ecological dynamics is the amount of heritable trait variation in a population. Variation is the raw material upon which natural selection acts (Fisher 1930; Dobzhansky 1937; Price 1972), but selection can, in turn, erode trait variation over time (Wright 1931, 1949). This may occur even if other processes such as mutation or gene flow can increase trait variation (Kimura 1991). Trait variation also can have important ecological consequences (Bolnick et al. 2011; Gibert and Brassil 2014). Indeed, trait variation has been theoretically shown to alter predator–prey (Gibert and Brassil 2014; Gibert and DeLong 2015) and eco‐evolutionary dynamics (Nuismer et al. 2005; Schreiber et al. 2011; Vasseur et al. 2011). Empirically, trait variation affects the reproductive rate of sockeye salmons (*Oncorhynchus nerka*, Greene et al. 2010), dispersal rate in threespine sticklebacks (*Gasterosteus aculeatus*, Laskowski et al. 2015), and tri‐trophic consumer–resource interactions in salt marshes (Hughes et al. 2015), among many other effects (Bolnick et al. 2011; Gibert et al. 2015). Thus, incorporating trait variation into eco‐evolutionary models is essential.

Current approaches to modeling eco‐evolutionary dynamics have important limitations. First, they require the specification of fitness functions that link parameters controlling ecological dynamics to traits (Jones et al. 2009; Schreiber et al. 2011; Vasseur et al. 2011). Typically, these fitness functions are based on the breeder's equation, following seminal work by Abrams et al. (1993) and Lande (1976), but other formulations are also possible (Lande 1979). While these approaches are biologically sensible, they nevertheless make mathematical assumptions about how underlying biological traits determine the value of the parameters controlling ecological dynamics and their evolution. Furthermore, fitness functions may change through time (Siepielski et al. 2009), making the assumption of a static fitness landscape unrealistic. Because the fitness function that links ecological parameters to evolving traits is such an important part of the formulation of eco‐evolutionary models, there is a need to move toward more direct ways of incorporating the relationship between underlying evolving traits, ecological parameters, and fitness.

Second, current approaches do not incorporate trait variation in a sufficiently realistic manner. Some classic works do not incorporate trait variation at all (Fussmann et al. 2003; Yoshida et al. 2003), while others keep it at a fixed level (Schreiber et al. 2011; Vasseur et al. 2011). Models that track the abundance of discrete morphs whose frequencies in the population change over time allow tracking of trait variance but do not show how changes in variation may influence the dynamics (e.g., Jones et al. 2009; Ellner and Becks 2011). Integral projection models also enable the tracking of trait variation (Easterling et al. 2000; Smallegange & Coulson 2013; Rees et al. 2014), but they do not consider multiple species and their interactions (Rees et al. 2014), making them unsuitable for studying eco‐evolutionary dynamics as of now, except for those occurring on the focal population. Other approaches model changes in trait variation given the selective pressures defined in a fitness function (Nuismer et al. 2005; Tirok et al. 2011). In this approach, variation is tied to the mean of the trait. This connection may be appropriate for some trait distributions (e.g., a normal distribution), but it does not allow for reduction of variance by selection against one or the other end of the trait distribution, preventing realistic loss of trait variation from influencing the ecological dynamics. Furthermore, this latter type of model uses the assumed fitness gradient to generate changes in trait variation (Nuismer et al. 2005; Tirok et al. 2011), bringing us back to the need to find a more direct formulation of fitness gradients. In short, current theory does not provide sufficient insight into how the erosion (through selection or genetic drift) or amplification (through recombination, mutation, or immigration) of variation may lead to different eco‐evolutionary scenarios and dynamics. Although modeling the ways in which trait variation may change over time clearly has been a long‐standing problem (Bull 1987; Steppan et al. 2002; Arnold et al. 2008), keeping realistic track of trait variation must be considered a cornerstone of the budding area of eco‐evolutionary dynamics.

**Figure 1:**
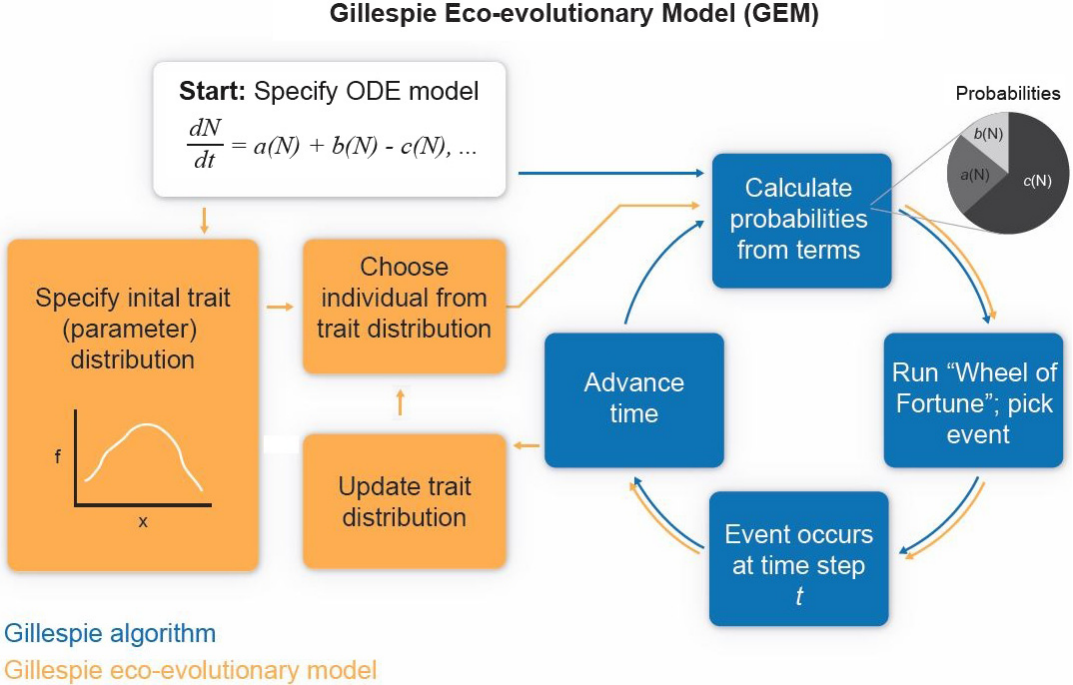
Schematic of how Gillespie eco‐evolutionary models work.

Here, we develop a new class of eco‐evolutionary model that incorporates and tracks the amount of heritable variation in multiple traits controlling ecological interactions while also eliminating the need for assumed fitness functions. This new type of model, called a Gillespie eco‐evolutionary model (GEM), involves adding a side‐loop to the standard Gillespie stochastic algorithm (Gillespie 1977) in which (1) both the mean and variance of a trait in the population may influence the dynamics; (2) fitness is determined by the effect that a particular parameter value has on birth and death rates of the evolving organism in the context of the model; and (3) offspring traits depend on the population variance and heritability of the trait (Fig. 1). We argue that GEMs are a powerful and general way of evaluating the effect of contemporary trait evolution on ecological dynamics for any process that can be modeled using ordinary differential equations (ODEs).

To illustrate how GEMs work and what insights we can gain from such an approach, we show several examples of eco‐evolutionary dynamics based on the classic Rosenzweig–MacArthur (RM) consumer–resource model (Rosenzweig and MacArthur 1963). We first describe how GEMs work and set up the RM model as a GEM. We then conduct three levels of analysis: (1) we run the GEM with and without heritable trait variation to show how its addition alters the consumer–resource dynamics relative to the standard, nonevolutionary dynamics; (2) we run the model allowing each parameter in the RM model to evolve in turn; and (3) we connect model parameters to a physical trait – body size – to illustrate trait‐parameter links and the potential for multiple functional consequences of trait evolution. Our results show how trait variation and its change over time due to selection and stochasticity can lead to different eco‐evolutionary outcomes, and we discuss how these results may change the way we study eco‐evolutionary dynamics.

## How GEMs Work

**Figure 2:**
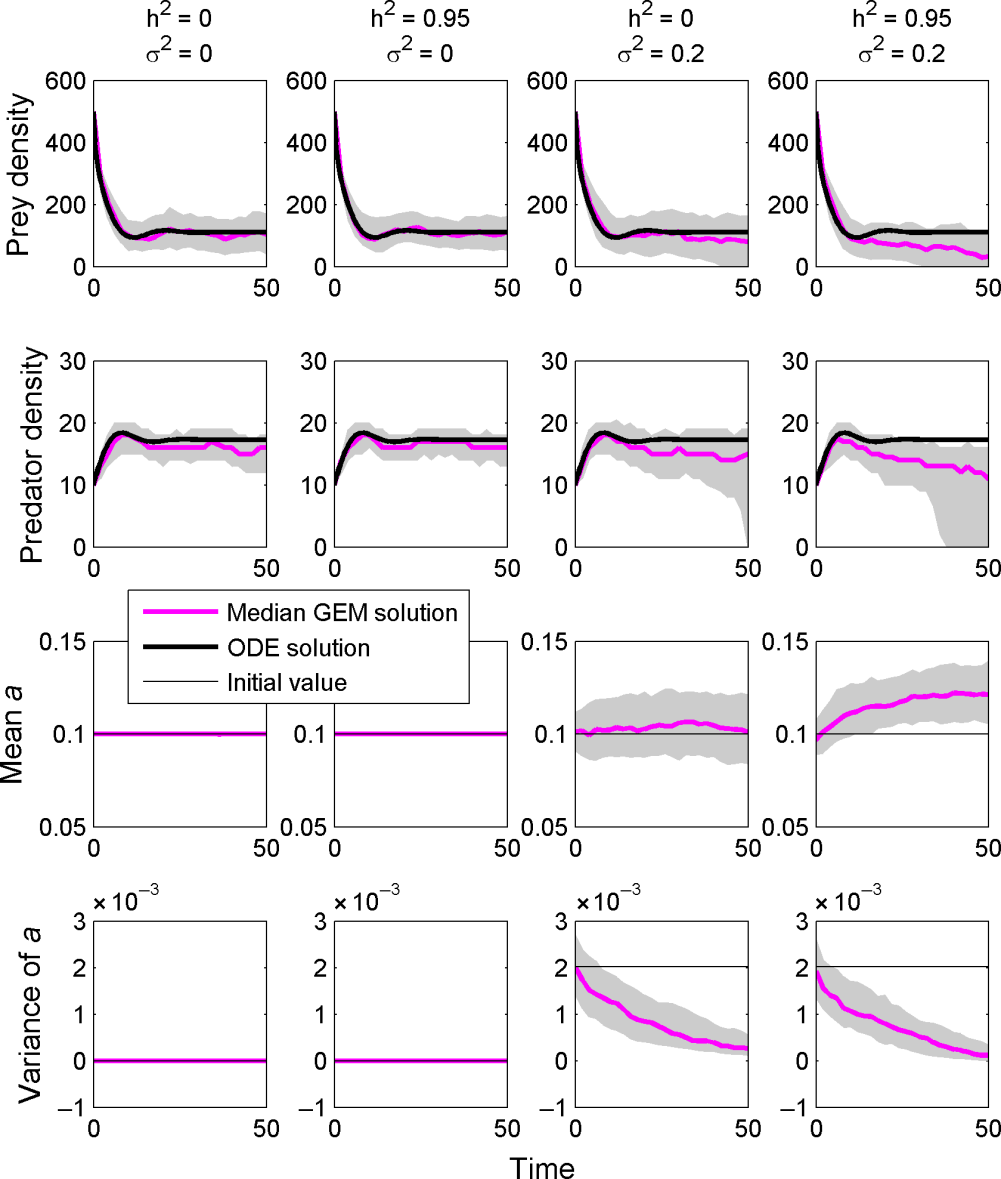
Four versions of the same GEM with different levels of heritability and trait variation. The model parameters used were *h *=* *0.01, *e *=* *0.01, *d *=* *0.1, *r *=* *2, *K *=* *500. The starting value for *a* was 0.1.

Gillespie eco‐evolutionary models use a Gillespie algorithm to stochastically simulate an ODE model (Gillespie 1977; Yaari et al. 2012). A Gillespie algorithm approximates a continuous‐time ODE with discrete time “events,” such as births, predation, and deaths for a population of size *N*. In the example in Figure 1, the different events that can happen at each time step are given by the model, and in this cartoon example, we have arbitrarily set the model terms as *a*(*N*), *b*(*N*), and *c*(*N*). To determine which event occurs, each term is divided by the sum of all terms. This can be visualized as a “wheel of fortune” where the sizes of the segments on the wheel are set by the relative magnitude of the terms (gray pie chart in Fig. 1). At each time step, the wheel is figuratively “spun,” and a location on the wheel is randomly chosen, determining which event occurs. The larger segments of the wheel are more likely to be selected, making those events more common in the simulation. The Gillespie algorithm advances time after each event through a random draw from an exponential distribution scaled to the number of individuals in the system. This process repeats until some time limit chosen by the user is reached. Typically, as long as stochastic extinctions are not common, the mean output of a Gillespie simulation converges to a standard numerical solution for the ODE (Fig. 2, top left).

A GEM is different than a standard Gillespie simulation in that instead of using all constant parameters, some or several parameters of interest are treated as heritable traits and given distributions that are allowed to change over time (Fig. 1). For each event, a value is randomly drawn from the current parameter distribution, and that value is used to set up the event probabilities (i.e., the size of the segments of the wheel of fortune depend on the value drawn). In this way, the likelihood of any event changes with the parameter drawn. After the parameter is chosen, the Gillespie algorithm proceeds as usual and time advances. In this process, one can treat the model parameter as a trait in and of itself or make the parameter a function of a physical trait such as body size. If the trait is a physical trait, then the trait distribution is set up for that trait, and the parameter is given by the trait‐parameter map (e.g., an allometry). This is akin to the phenotype‐demography map of Coulson et al. (2006), but it links traits to parameters whether they are demographic in nature (e.g., death rate) or not (e.g., functional responses).

In a GEM, when the event is a death, the current value of the parameter is removed from the parameter distribution. The consequence is that any parameter value that is relatively likely to lead to a death tends to be removed, causing the distribution to move away from that value. Whether a particular parameter alters the likelihood of a death depends on the model and the role of that parameter in setting the death rate. Similarly, when an individual is born, the new individual takes on a value that is similar to its parent's (i.e., the value drawn at the beginning of the current iteration), depending on how heritable it is, and that new value is added to the population, thus altering the distribution of the trait. In this way, values that are likely to lead to births tend to become more represented in the population. As with deaths, whether a particular parameter alters the likelihood of a birth depends on the model and the role of that parameter in setting the birth rate. The parameter distribution therefore moves in a direction determined by the direct fitness consequences of the parameter values present in the population at a given time and in the context of the interactions specified by the underlying model, and therefore, no fitness function needs to be included. The output of a GEM is an approximate solution to the system of ODEs where both population sizes and a distribution of traits (parameters or physical traits) may change through time.

## An example

We developed a GEM for the RM model (see Data S1 for MATLAB code). This model is a set of coupled differential equations for the dynamics of a consumer population (*C*) consuming a resource population (*R*):

(1) $$\frac{dR}{dt}=rR\left(1-\frac{R}{K}\right)-\frac{aRC}{1+ahR}$$

(2) $$\frac{dC}{dt}=\frac{eaRC}{1+ahR}-dC$$

In this model, *r* is the resource intrinsic growth rate, *K* is the resource carrying capacity, *a* is the area of capture (also known as attack rate or attack efficiency), *h* is handling time, *e* is the efficiency of converting resources into new consumers, and *d* is the background death rate for the consumer. The events in this model are resource births, *rR*.d*t*, density‐dependent resource deaths, *rR* ^2^/*K*.d*t*, consumer‐caused resource deaths, *aR*/(1 + *ahR*).d*t*, consumer births, *eaR*/(1 + *ahR*).d*t*, and consumer deaths, d*C*.d*t*. These events have a certain likelihood of occurring given the parameter values.

An interesting question that needs to be answered when building a GEM is what assumptions should be made about the distribution of the parameter that one is interested in tracking. Many parameters in a model like the RM model are likely to be driven by the additive effects of multiple physical or behavioral traits, some of which may have additive genetic variance. It is reasonable then to think that the distribution of the parameters within a population should be approximately normal. Yet the parameters cannot be negative, so they also are likely to have at least some tendency to skew positively. Although a variety of options may be appropriate, in this analysis, we assume that all parameters have a starting variance of 0.2 times the initial trait mean and a slight right skew. These assumptions provide realistic distributions that do not cross zero (Figure S1), but other distributions are also possible, including empirically determined distributions when available.

When a birth occurs in a GEM, the parameter value of the new individual is randomly drawn from a distribution of potential values (an “offspring sampling distribution”) determined by the parent's parameter (i.e., the value of the parameter that was randomly chosen prior to the birth event) and the heritability of that parameter (*h* ^2^; Figure S2) (this is inherently an asexual form of reproduction, although approaches to approximating sexual reproduction could be incorporated). Theoretically, traits can range from being not heritable at all to being perfectly heritable. To accommodate this range, we determined the variance of the distribution from which the offspring trait value was sampled as (1 − *h* ^2^)*σ* ^2^, where *σ* ^2^ is the current variance of the population parameter distribution. The mean of the distribution from which offspring trait values were sampled was given as *h* ^2^(*x*_parent_ − *x*_mean_) + *x*_mean_. Thus, when *h* ^2^ = 0, the mean of this sampling distribution is the current population mean and the variance is the same as the whole parameter distribution, such that the offspring could come from anywhere in the current distribution. The more heritable the trait, the more the offspring will tend to look like the parent, and when *h* ^2^ = 1, the offspring is identical to the parent.

## Three levels of analysis

### GEMs with and without heritable trait variation

We first ran the GEM under four scenarios with heritability at 0 or 0.95 (not 1 because it is highly unlikely for any trait to be perfectly heritable) and starting parameter variance at 0 or 0.2*parameter mean. These four scenarios depict the range of possible outcomes from a standard Gillespie simulation to a GEM for a highly heritable trait and show the independent consequences of adding variance and heritability to the simulation. In this first example, the parameter of interest was the area of capture from the functional response (eq. (1)). This parameter here is designated a predator trait.

### GEMs for each model parameter

We then set the heritability at 0.75 and ran the GEM for each of the six parameters in the RM model in turn (that is, each simulation allowed one parameter to change at a time). The area of capture, handling time, conversion efficiency, and death rate parameters were designated predator traits, and the intrinsic growth rate and carrying capacity were designated as prey traits. Although some of these parameters may reflect contributions from both predator and prey (for example, both prey and predator velocities influence the area of capture; Aljetlawi et al. 2004), we assign each parameter to one population for simplicity in this illustration. Furthermore, some parameters, such as carrying capacity, clearly emerge from the interactive effects of physical traits and environmental inputs. We allow each parameter to evolve, including carrying capacity, given that each has at least some genetically based trait or set of traits that influence the parameter. Each simulation involved 200 runs, and we extracted the median and inner 50% quantile range of the runs to display. Finally, we calculated the level of parameter variation and the change in the median trait through time to assess the dependence of parameter change on the level of variance using general linear models.

### GEMs for body size with trait‐parameter maps

Finally, we set up the GEM with predator body size as the evolving trait, because body size is linked to many parameters in consumer–resource models (DeLong and Vasseur 2012a). Here, we start body size at the approximate average cell volume of the predatory ciliate *Didinium nasutum* (~1 × 10^5^ *μ*m^3^) (DeLong et al. 2014). We made a trait‐parameter map to link cell volume to the area of capture parameter (*a *=* *1.1 × 10^−7^ *M* ^1^) and the conversion efficiency (*e* = 2.16 *M* ^−0.5^) based on the allometric relationships for protists given in DeLong et al. (2015), where *M *= cell volume of the predator. We initialized the trait distribution in the same way as the parameter distributions above, and each time a particular value of cell volume was drawn, the trait‐parameter maps specified the parameters to be used in the next time step of the GEM. We ran these simulations with cell volume evolving only when connected to area of capture, only when connected to conversion efficiency, and when connected to both parameters simultaneously. Each simulation involved 200 runs, and we extracted the median and inner 50% quantile range of the runs to display.

## Results

### GEMs with and without heritable trait variation

Our GEM for the RM model clearly shows the eco‐evolutionary consequences of allowing model parameters to take on distributions and be heritable. When both the standard deviation and the heritability are zero, the output of a GEM is equivalent to a standard Gillespie simulation and reproduces well a standard numerical solution to the ODE (Fig. 2, left column). Adding heritability to the trait does not by itself cause any changes to this outcome as there is no variation in the parameter (Fig. 2, second column). Adding variation alone to the model produced changes in the model output (Fig. 2, third column). Overall, there was an increase in the variation of the simulation outcomes, which is a consequence of having variation in the parameter. Due to the increased variation of the population trajectories, there was increased extinction, and the median trajectory declined slightly through time. Although the parameter showed little change, the stochastic progression of the model eroded the parameter variance through time. Finally, including both variance and heritability in the model (Fig. 2, right column) caused shifts in the dynamics, the mean parameter value, and the level of variance.

### GEMs for each model parameter

**Figure 3:**
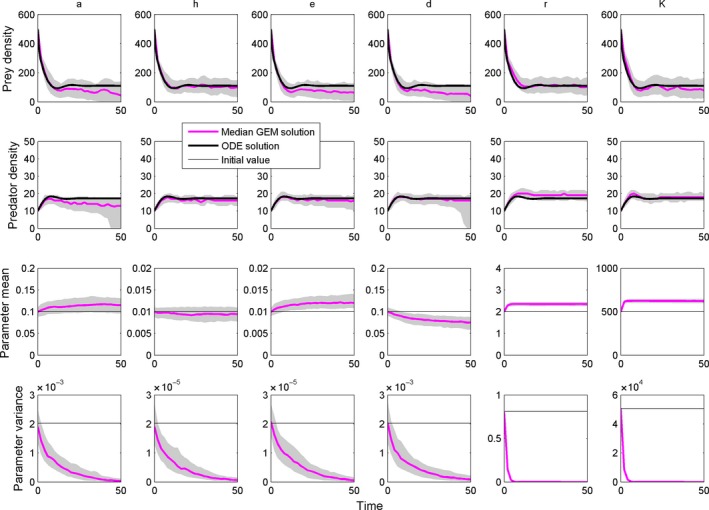
The Rosenzweig–MacArthur model solved with GEMs for each model parameter. Starting parameters were the same as in Figure 2.

When allowed to evolve in the GEM, all of the parameters except for the handling time showed changes in magnitude and variance that led to population dynamics that are different from those predicted by the nonevolutionary RM model (Fig. 3). As expected, the variance of all parameters was eroded through time, regardless of the direction of selection or the lack thereof (Fig. 3). More precisely, the variance was reduced on both sides of the distribution until there was no variance left (see Figure S3 for an example with the resource intrinsic rate of growth).

**Figure 4:**
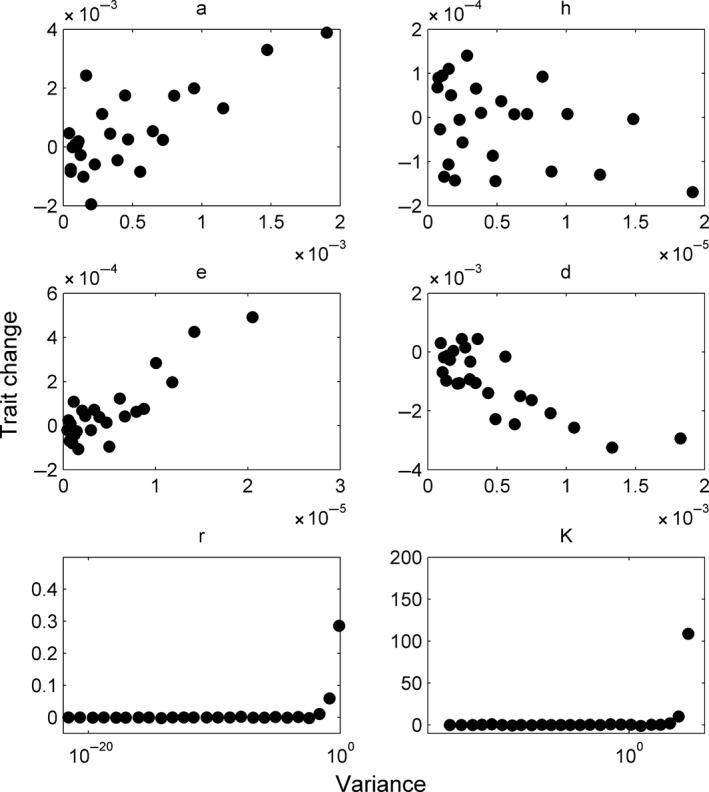
For most parameters, the rate of change in the trait (parameter) is positively related to the amount of variance still present in the population. Note that for *d* (consumer death rate), the trait is declining more quickly as variance increases. The parameter *h* (handling time) did not change much during the simulations, and so unsurprisingly its rate of change was not related to the variance.

As the variance in the parameter distributions decreased through time, so did the rate of trait change, for all traits except for the handling time, which did not evolve (Fig. 4). Thus, through either selection, drift or both, the decrease in the parameter distribution's variance tended to bring trait changes and eco‐evolutionary dynamics to a halt. For each unit increase in variance, *a* increased by 1.43 (±0.43 SE), *e* by 16.24 (±4.95 SE), *r* by 0.40 (±0.01 SE), *K* by 0.0006 (±0.00018 SE), and *d* decreased by 1.96 (±0.44 SE). There are arguments against using traditional statistical tests in analyses of simulations, due to the effect of arbitrarily high sample sizes (White et al. 2014), so we present the relationships between parameter variance and rate of trait change in Figure 4 but do not include significancy levels of the slopes reported above.

### GEMs for body size with trait‐parameter maps

**Figure 5:**
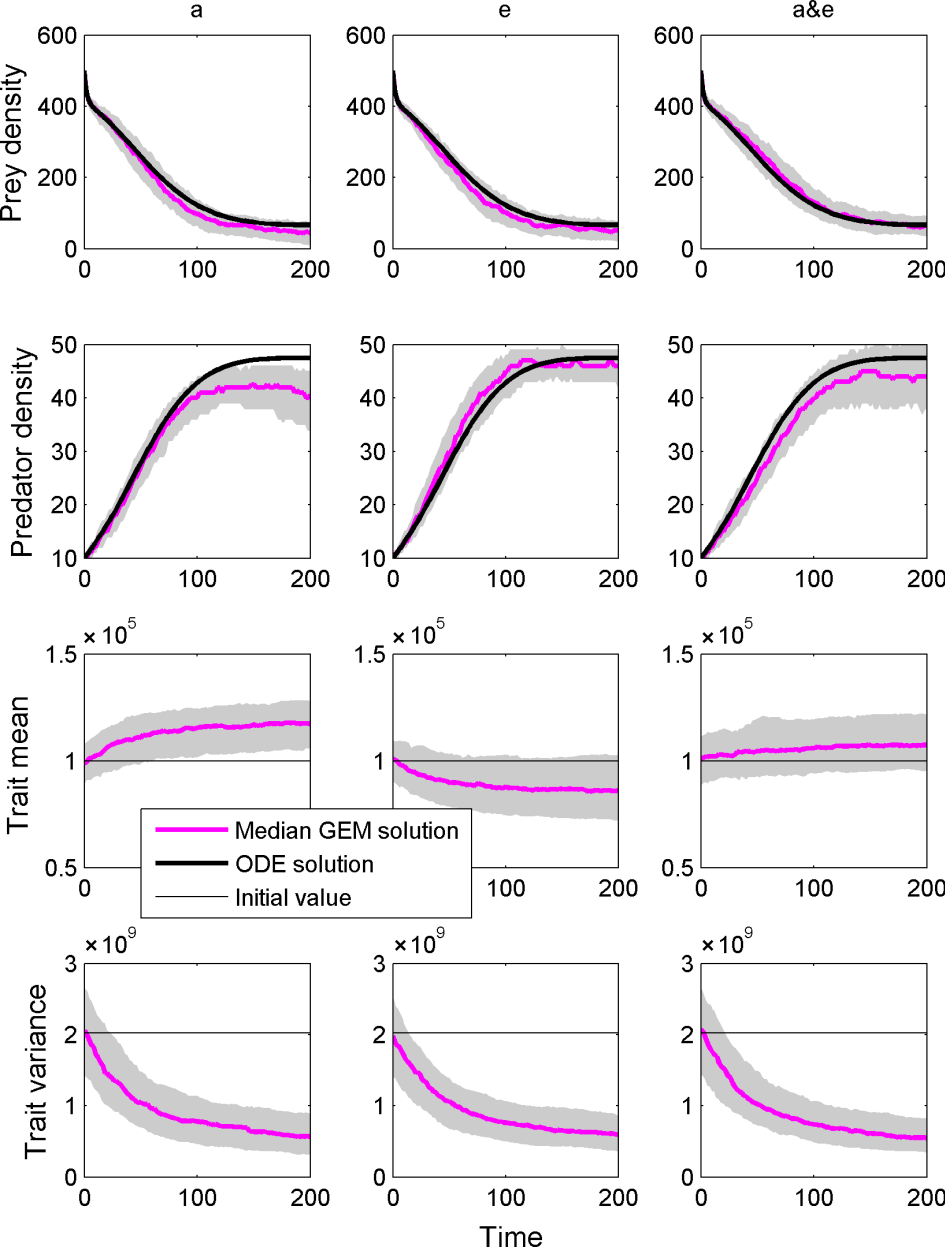
The Rosenzweig–MacArthur model solved with GEMs with body size as the evolving trait. Body size was linked first to the area of capture parameter (left column), then to the conversion efficiency parameter (middle column), and finally to both parameters (right column). Body mass was mapped to area of capture and conversion efficiency using allometries from DeLong et al. (2015), and the remaining parameters were *h *=* *0.005, *d *=* *0.005, *r *=* *0.6, *K *=* *500.

When linked to area of capture, body mass increased through time, but when linked to conversion efficiency, body mass decreased through time (Fig. 5). When linked to both parameters, body mass increased but to a lesser extent than it increased when linked only to area of capture. As with the evolution of the parameters, heritable trait variation is eroded through time, reducing the rate of trait evolution and limiting the degree of eco‐evolutionary dynamics in the long run.

## Discussion

Gillespie eco‐evolutionary models represent a major step forward in eco‐evolutionary modeling because they incorporate the effects of trait variation and fitness into ecological dynamics generated by multiple interacting species without the need for assumptions about fitness gradients. Our example with the RM consumer–resource model shows that when sufficient heritable trait variation occurs, some parameters will tend to increase (area of capture, conversion efficiency, intrinsic rate of growth, carrying capacity), some will tend to decrease (predator mortality rate), and some will not change at all (handling time). These shifts are all consistent with expectations about how each parameter influences the fitness of the consumer or the resource. For example, a higher value for the area of capture (*a*) parameter will lead to more food acquisition and more births for a predator bearing a high area of capture, so the trait should increase through time.

The changes in the parameters that are seen in Figure 3 reflect, in essence, the fitness landscape dictated by the RM model. By allowing the parameters to evolve as traits, we show how the model sets up opportunities to increase fitness for the consumer or the resource. For example, increasing the conversion efficiency increases birth rates, increasing fitness for the consumer. Any physical trait that influences the conversion efficiency parameter, then, can evolve by following this path. Our analysis with body mass (Fig. 5) shows that a smaller body mass increases conversion efficiency (by making the cost of an offspring smaller), so body mass evolves to a smaller size to increase fitness. In contrast, larger size increases foraging rates by increasing the area of capture, so body mass evolves to a larger size to increase fitness. The speed of this process depends on the actual functional link between the trait and the parameter, the amount of heritable variation, and the model itself.

Our body size (cell volume) analysis also reinforces the potential for links or trade‐offs among parameters (Yoshida et al. 2003; Becks et al. 2012; DeLong and Vasseur 2012b, 2013). Body mass is linked to conversion efficiency and area of capture with opposing fitness effects. We argue that this is a case of “ecological pleiotropy,” wherein a physical trait (rather than a gene) influences more than one ecological process (such as area of capture and conversion efficiency) that is linked to fitness. Similarly, some traits may influence the fitness of more than one species in an ecological interaction. For example, prey defense traits might influence both prey and predator fitness (Jeschke and Tollrian 2000). Co‐eco‐evolutionary dynamics therefore may arise in a GEM if the parameters can be matched to different traits of the different interacting populations in the system.

All of the changes in mean parameters were also accompanied by changes in their associated variance, which leads to dynamics that are qualitatively different from the prediction of models that do not consider changes in variance. The simulations show that the changes in traits slow down as the level of variance diminishes through time (Figs. 3, 4, 5), whether due to selection or random erosion of variance or both. Thus, eco‐evolutionary dynamics are more pronounced at the beginning of the simulations, and although they come to a halt, there remains a longer‐term shift in the parameters that cause the dynamics to remain different even after evolution stops. Alternatively, the actual changes in the parameters through time could be responsible for changes in the magnitude of selection and also contribute to the slowdown in the rate of trait change. The ability of GEMs to track and incorporate current trait variation into the dynamics reveals patterns that would be hard to detect if we utilized a constant level of variance throughout. This illustrates both the importance of tracking variance in eco‐evolutionary models and the efficacy of GEMs in accomplishing this crucial goal.

These results, however, call attention to the question of how variance should be increased or maintained in a GEM, or indeed any other eco‐evolutionary model (Smallegange and Coulson 2013), especially given how trait variation also affects the pace at which traits evolve (Figs. 3, 4, 5). It is also possible to consider other factors leading to higher trait and parameter variance through time, by allowing for mutations when drawing offspring distributions, or to boost trait variation when, for example, a parthenogenetic organism like *Daphnia* switches to sexual reproduction under certain scenarios or when new genetic variants immigrate to the population (Tirok et al. 2011). GEMs provide the framework for dealing with such complexities, but how to mathematically incorporate these increases in variance may vary by system.

In summary, GEMs are a novel and important addition to the eco‐evolutionary modeling toolbox. Although they represent a natural and direct characterization of selection in an ecological context, GEMs are still models, and so they should be compared to empirical data to see how well they perform (along with competing modeling approaches as well). GEMs can be developed for any ODE model, including predator–prey, parasite–host, epidemiological, energy budget, and many other types of models. GEMs provide all the benefits of traditional ODE models, stochastic models, integral projection models, and individual‐based models with fewer assumptions, inherent fitness effects, and less computational demand. We envision a slew of new insights into eco‐evolutionary dynamics arising from the use of GEMs to study a wide variety of eco‐ and co‐ecoevolutionary dynamics.

## Conflict of Interest

The authors declare no conflict of interest.

## Supporting information


**Figure S1**. Depiction of the shape of initial parameter distributions.
**Figure S2**. Examples of how offspring sampling distributions are calculated.
**Figure S3**. Distribution of the parameter r (prey intrinsic growth rate) through time for a single simulation.

**Data S1**. Matlab scripts.
