# Supplementary material for: Gillespie eco‐evolutionary models (GEMs) reveal the role of heritable trait variation in eco‐evolutionary dynamics
Source: Ecol Evol. 2016 Jan 18;6(4):935–45. doi: 10.1002/ece3.1959 (PMC4761774; doi:10.1002/ece3.1959)

**Supplemental Figures for Gillespie eco-evolutionary models (GEMs) reveal the role of heritable trait variation in eco-evolutionary dynamics**

John P. DeLong and Jean P. Gibert

**Figure S1**. Depiction of the shape of initial parameter distributions. These distributions have a slight right skew and a variance that is 0.2 times the mean. All distributions cover about a two-fold range in magnitude and robustly do not cross zero into negative values.


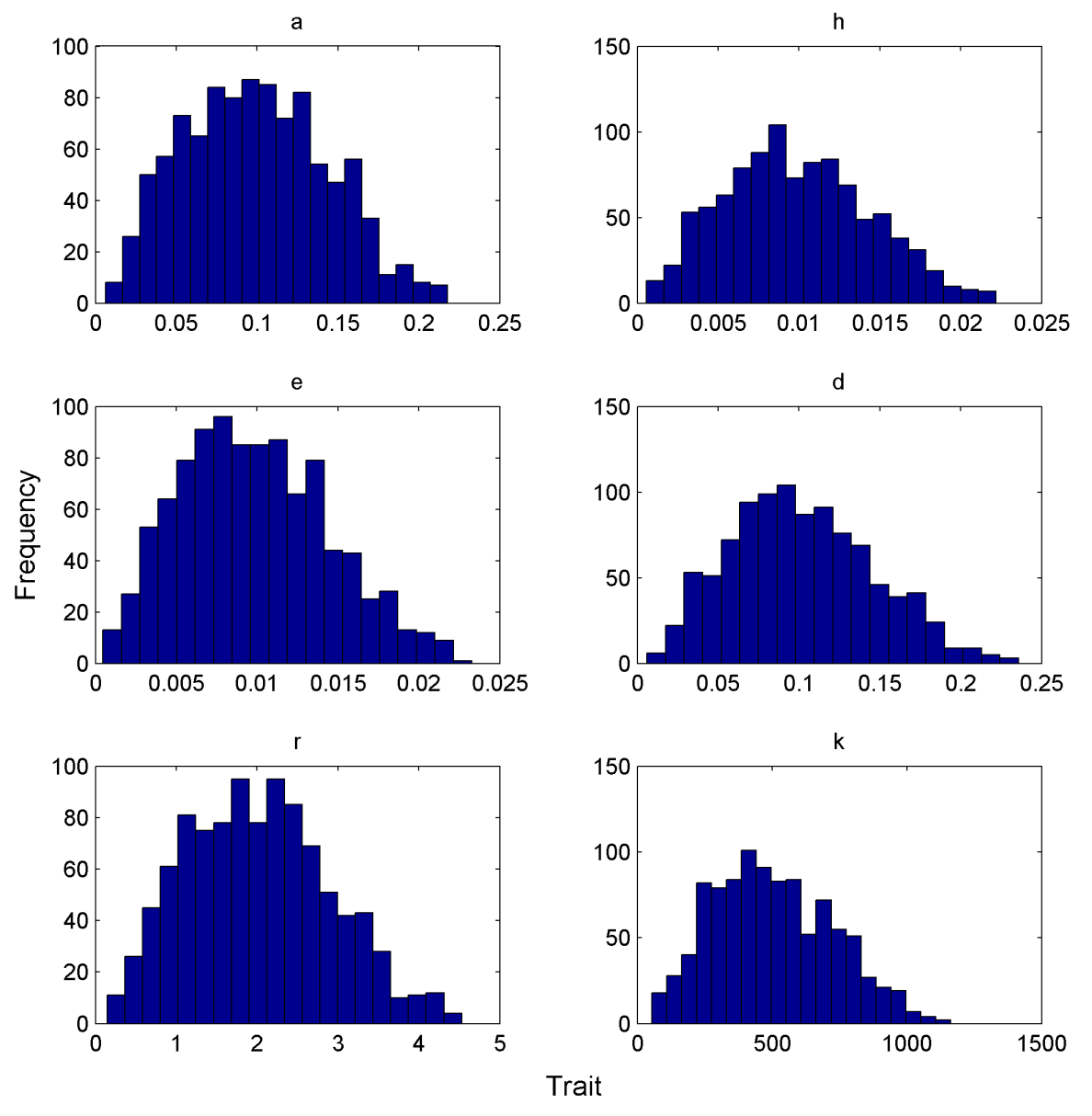


**Figure S2**. Examples of how offspring sampling distributions are calculated. The offspring sampling distribution is the distribution from which the trait of a new individual is randomly drawn. It is closer to the parent the higher the heritability and the lower the population-level trait variation. The black lines show a current population distribution and mean. In scenario 1 (green), the parent has a trait of 900 and the heritability is 0.95. This generates an offspring sampling distribution in the green area, which is very close to the parent as expected. In scenario 2 (blue), the parent’s trait is 300 and the heritability is 0.6. As expected, the offspring trait has more chance of being unlike the parent given the low heritability.


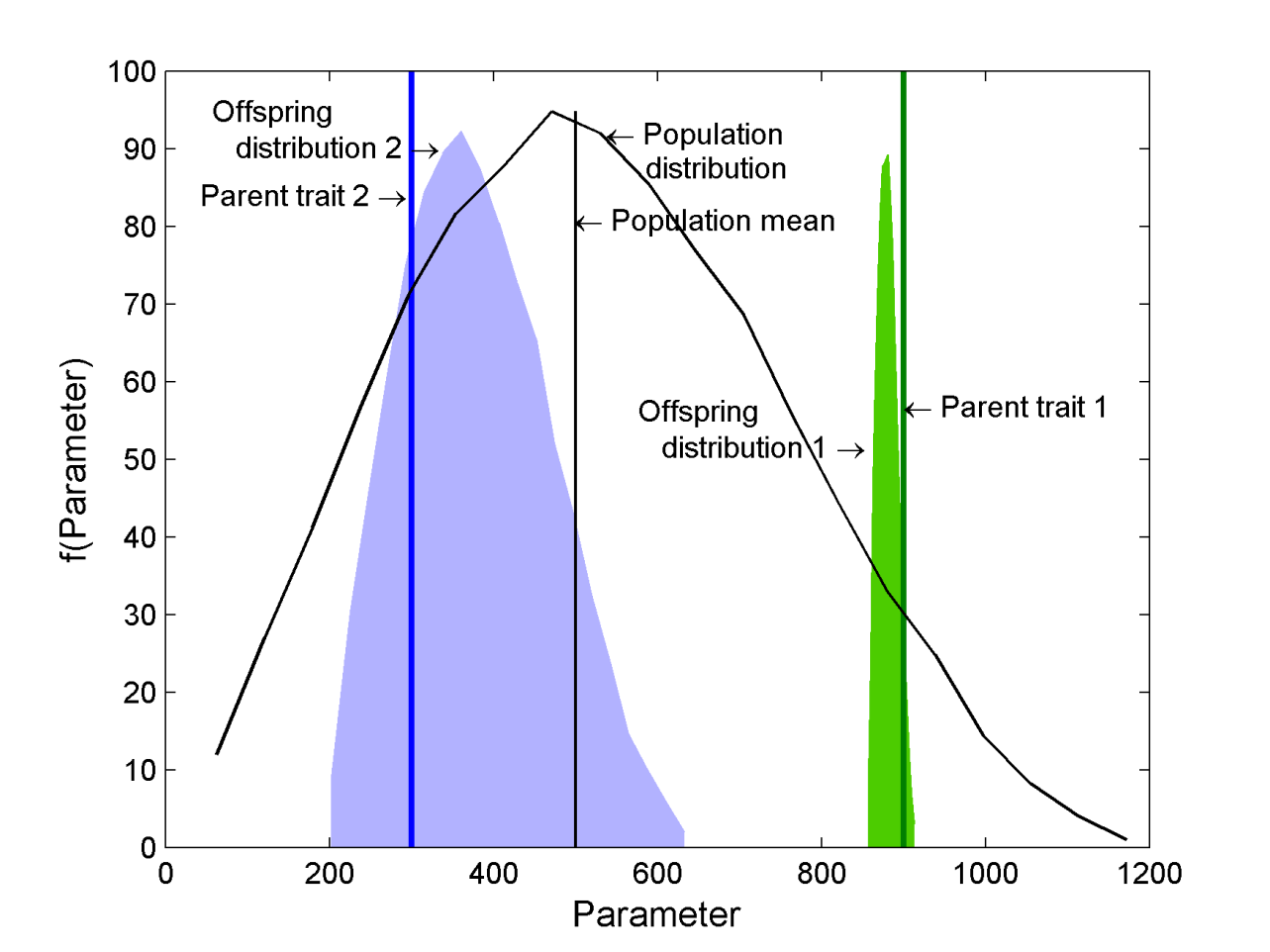


**Figure S3**. Distribution of the parameter r (prey intrinsic growth rate) through time for a single simulation.


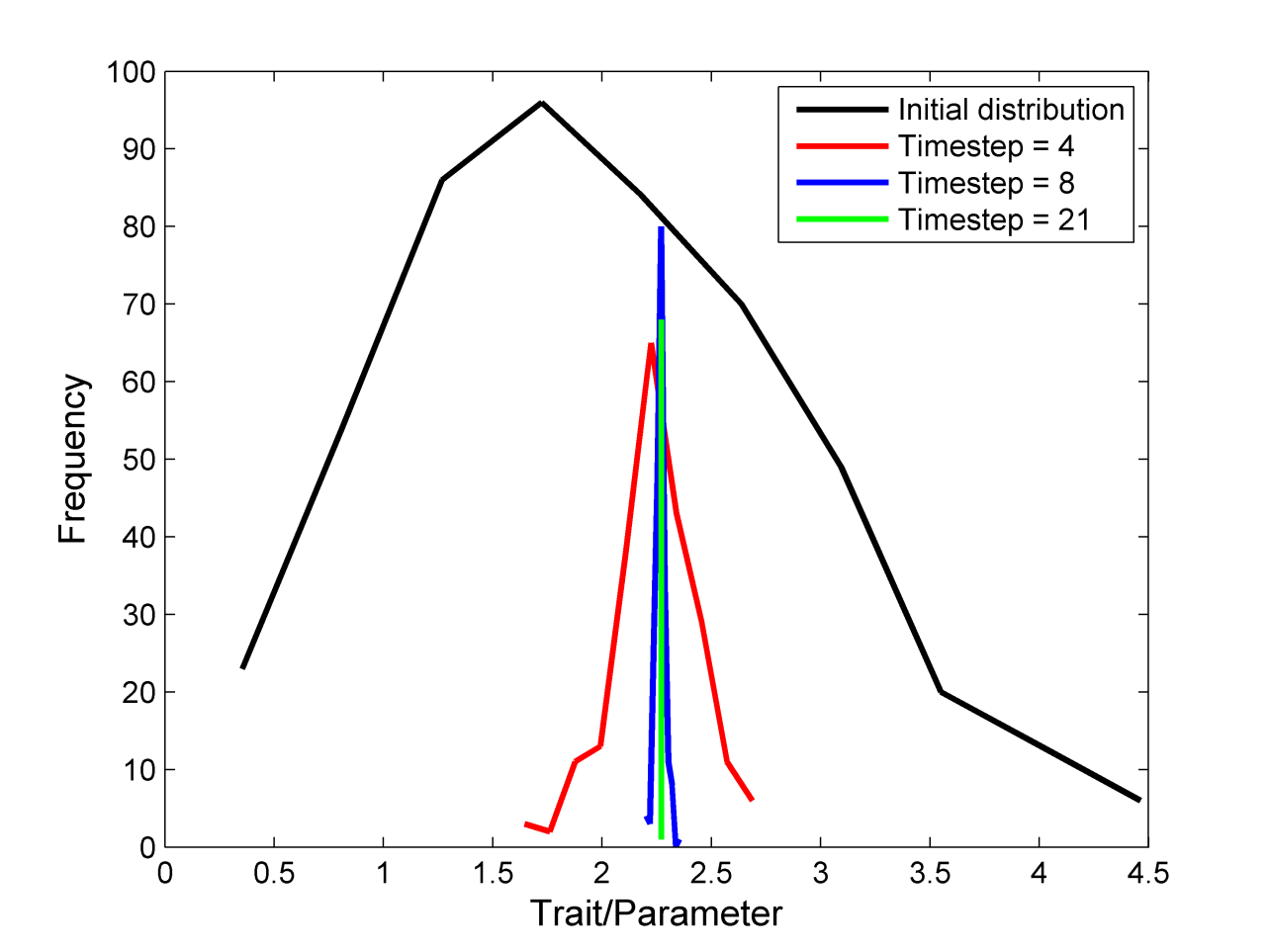

Supplement: Supplementary file 1 — Figure S1. Depiction of the shape of initial parameter distributions. Figure S2. Examples of how offspring sampling distributions are calculated. Figure S3. Distribution of the parameter r (prey intrinsic growth rate) through time for a single simulation. [file ECE3-6-0935-s001.docx]
