# Supplementary material for: Gillespie eco‐evolutionary models (GEMs) reveal the role of heritable trait variation in eco‐evolutionary dynamics
Source: Ecol Evol. 2016 Jan 18;6(4):935–45. doi: 10.1002/ece3.1959 (PMC4761774; doi:10.1002/ece3.1959)
Supplement: Supplementary file 2 — Data S1. Matlab scripts. [file ECE3-6-0935-s002.docx]

Supplemental text. Matlab scripts.

Code for Figures 3 and 4.

clear; clc; clf;

tic

r = 2; % specify initial ODE parameters

k = 500;

h = 0.01;

e = 0.01;

d = 0.1;

a = 0.1;

h_2 = 0.75; % define level of heritability

t_max = 50; % time span

prey_init = k; % initial prey density

pred_init = 10; % initial pred density

%% run standard solver on differential Mac Ros equation

y0 = [prey_init pred_init]; % initial prey and predator densities

tspan = [0 t_max]; % start end times

ode = @(t,y) MR_model(t,y,r,k,a,h,e,d); % compile function and call

[t1,y1] = ode45(ode, tspan, y0); % return time and population density vectors

%% Gillespie algorithm

parameters = {'a','h','e','d','r','K'}; % group parameters

parameters2 = [a h e d r k];

std_scalar = [0.45 0.45 0.45 0.45 0.45 0.45];

for j = 1:6

parameter_to_use = parameters2(j);

current_param = parameters(j);

num_replicates = 200; % number of simulations

stand_times = 0:2:t_max; % standardized time steps for storing time series

num_time_steps = length(stand_times);

n_stand = nan(num_replicates,num_time_steps); % preallocate matrix for standardized population size

p_stand = nan(num_replicates,num_time_steps);

x_stand = nan(num_replicates,num_time_steps);

x_var_stand = nan(num_replicates,num_time_steps);

for i = 1:num_replicates % start Gillespie algorithm

% preallocate for whole time series

n = zeros(1,1e5); % 1e5 is just a large number to ensure it's long enough

p = zeros(1,1e5);

t = nan(1,1e5);

x_mean = nan(1,1e5);

x_var = nan(1,1e5);

% define initial states

t(1) = 0; % initial time

n(1) = prey_init; % initial prey population size

p(1) = pred_init; % initial predator population size

% create initial distribution for parameter

rng('shuffle'); % change random number seed

if j < 5 % for j = 1 to 4, x's belong to predator

x_dist_init = pearsrnd(parameter_to_use,std_scalar(j)*parameter_to_use,0.3,2.5,pred_init,1); % specify initial %distribution of traits

else % for j = 5 or 6, x's belong to prey

x_dist_init = pearsrnd(parameter_to_use,std_scalar(j)*parameter_to_use,0.3,2.5,prey_init,1); % specify initial %distribution of traits

end

while sum(x_dist_init<0) > 0 % if there is a negative in x_dist_init, replace it

x_dist_init(x_dist_init<0) = pearsrnd(parameter_to_use,std_scalar(j)*parameter_to_use,0.3,2.5,sum(x_dist_init<0),1)

end

x_dist = x_dist_init; % reset parameter distribution at the start of each simulation

x_mean(1) = mean(x_dist); % initial mean parameter

x_var(1) = var(x_dist); % initial variance in parameter

count = 1; % start counter to index steps while inside loop

while t(count) < t_max

if j > 4 && n(count) > 0 || j < 5 && p(count) > 0 % as long as population size is > 0, pick another individual

R = randi(length(x_dist),1); % randomly choose individual from the vector

x_next = x_dist(R); % pick the parameter for that individual

else x_next = 0;

end

% set up rates of each possible event, given by ODE in MR_model.m

% birth rate of prey

if j == 5 % at j = 5, evolving parameter is r

b_n = x_next*n(count);

else

b_n = r*n(count); % otherwise r is fixed

end

% natural death rate of prey

if j == 5 % at j = 5, evolving parameter is r

d_n_1 = x_next*n(count)^2/k;

elseif j == 6 % at j = 6, evolving parameter is k

d_n_1 = r*n(count)^2/x_next;

else

d_n_1 = r*n(count)^2/k; % otherwise r and k are fixed

end

% mortality rate from predation

if j == 1

d_n_2 = x_next*n(count)*p(count)/(1+x_next*h*n(count)); % at j = 1, evolving parameter is a

elseif j == 2

d_n_2 = a*n(count)*p(count)/(1+a*x_next*n(count)); % at j = 2, evolving parameter is h

else

d_n_2 = a*n(count)*p(count)/(1+a*h*n(count)); % otherwise a and h are fixed

end

% predator birth rate

if j == 3;

b_p = x_next*d_n_2; % at j = 3, evolving parameter is e

else

b_p = e*d_n_2; % otherwise e is fixed

end

% predator death rate

if j == 4

d_p = x_next*p(count); % at j = 4, evolving parameter is d

else

d_p = d*p(count); % otherwise d is fixed

end

% sum the events to make wheel of fortune

sum_events = b_n + d_n_1 + d_n_2 + b_p + d_p;

r_num = rand*sum_events; % pick event

% now choose actual events

if (r_num < b_n) % choose birth of prey

n(count+1) = n(count) + 1; % add a prey

p(count+1) = p(count); % hold predator population constant

if j > 4

off_std = std(x_dist)*(1 - h_2); % specify offspring std given heritability h_2

x_parent = h_2*(x_next - mean(x_dist)) + mean(x_dist);

x_dist(length(x_dist)+1) = pearsrnd(x_parent,0.9*off_std,0.3,2.5,1,1); % add new individual to distribution

while x_dist(end) < 0 % if the trait ends up negative, pick again

x_dist(length(x_dist)) = pearsrnd(x_parent,0.9*off_std,0.3,2.5,1,1);

x_dist(end)

end

x_mean(count+1) = mean(x_dist); % calculate new mean

x_var(count+1) = var(x_dist); % calculate new variance

else

x_mean(count+1) = x_mean(count); % hold trait mean

x_var(count+1) = x_var(count); % hold trait variance

end

elseif (r_num >= b_n) && (r_num < b_n + d_n_1) % choose natural death of prey

n(count+1) = n(count) - 1; % take away a prey

p(count+1) = p(count); % hold predator population constant

if j > 4

x_dist = x_dist([1:R-1,R+1:end]); % reduce dist by lost individual

x_mean(count+1) = mean(x_dist); % calculate new mean

x_var(count+1) = var(x_dist); % calculate new variance

else

x_mean(count+1) = x_mean(count); % hold trait mean

x_var(count+1) = x_var(count); % hold trait variance

end

elseif (r_num >= b_n + d_n_1) && (r_num < b_n + d_n_1 + d_n_2) % choose prey death from predation

n(count+1) = n(count) - 1; % take away a prey

p(count+1) = p(count); % hold predator population constant

if j > 4

x_dist = x_dist([1:R-1,R+1:end]); % reduce dist by lost individual

x_mean(count+1) = mean(x_dist); % calculate new mean

x_var(count+1) = var(x_dist); % calculate new variance

else

x_mean(count+1) = x_mean(count); % hold trait mean

x_var(count+1) = x_var(count); % hold trait variance

end

elseif (r_num >= b_n + d_n_1 + d_n_2) && (r_num < b_n + d_n_1 + d_n_2 + b_p) % choose predator birth

n(count+1) = n(count); % hold prey population constant

p(count+1) = p(count) + 1; % add an individual to p

if j < 5

off_std = std(x_dist)*(1 - h_2); % specify offspring std given heritability h_2

x_parent = h_2*(x_next - mean(x_dist)) + mean(x_dist);

x_dist(length(x_dist)+1) = pearsrnd(x_parent,0.9*off_std,0.3,2.5,1,1); % assign new individual a trait

while x_dist(end) < 0 % if the trait ends up negative, pick again >> would like to fix this!!!!

x_dist(length(x_dist)) = pearsrnd(x_parent,0.9*off_std,0.3,2.5,1,1);

x_dist(end)

end

x_mean(count+1) = mean(x_dist); % calculate new mean of a

x_var(count+1) = var(x_dist); % calculate new variance of a

else

x_mean(count+1) = x_mean(count); % hold mean value

x_var(count+1) = x_var(count); % hold variance value

end

elseif (r_num >= b_n + d_n_1 + d_n_2 + b_p) && (r_num <= b_n + d_n_1 + d_n_2 + b_p + d_p) % choose predator death

n(count+1) = n(count); % hold prey population constant

p(count+1) = p(count) - 1; % take away a predator

if j < 5

x_dist = x_dist([1:R-1,R+1:end]); % reduce dist by lost individual

x_mean(count+1) = mean(x_dist); % calculate new mean

x_var(count+1) = var(x_dist); % calculate new variance

else

x_mean(count+1) = x_mean(count); % hold mean value

x_var(count+1) = x_var(count); % hold variance value

end

elseif r_num == 0 % added this because when n = p = 0, r_num cannot be less than any rate

n(count+1) = n(count); % keep n the same

p(count+1) = p(count); % keep p the same

x_mean(count+1) = x_mean(count); % hold mean value

x_var(count+1) = x_var(count); % hold variance value

end

t(count+1) = t(count) + exp(-1/sum_events)/sum_events;

count = count+1;

end

% figure(1); % plot each individual time series as you go

% %--- only do this if you want to see each curve b/c it slows down

% subplot(4, 6, j); hold on;

% plot(t(1:count),n(1:count),'-','Color',[0.5 0.5 0.5]);

% xlim([0 t_max]);

% subplot(4, 6, j+6); hold on;

% plot(t(1:count),p(1:count),'-','Color',[0.5 0.5 0.5]);

% xlim([0 t_max]);

% subplot(4, 6, j+2*6); hold on;

% plot(t(1:count),x_mean(1:count),'-','Color',[0.5 0.5 0.5]);

% xlim([0 t_max]);

% subplot(4, 6, j+3*6); hold on;

% plot(t(1:count),x_var(1:count),'-','Color',[0.5 0.5 0.5]);

% xlim([0 t_max]);

% find standardized times and corresponding densities (need for ci's)

for q = 1:num_time_steps

val = stand_times(q); %value to find

tmp = abs(t-val);

[idx idx] = min(tmp); %index of closest value

closest = t(idx); %closest value

n_stand(i,q) = n(idx); % prey at standard time

p_stand(i,q) = p(idx); % pred at standard time

x_stand(i,q) = x_mean(idx); % mean a at standard time

x_var_stand(i,q) = x_var(idx); % var a at standard time

end

end

% calculate ci's for time series

upper_ci_level = 75; % choose ci levels

lower_ci_level = 25; % choose ci levels

% prey abundance

test(:,:) = n_stand(:,:);

ci_prey_up = prctile(test,lower_ci_level);

ci_prey_down = prctile(test,upper_ci_level);

median_prey = prctile(test,50);

% predator abundance

test(:,:) = p_stand(:,:);

ci_pred_up = prctile(test,lower_ci_level);

ci_pred_down = prctile(test,upper_ci_level);

median_pred = prctile(test,50);

% parameter

test(:,:) = x_stand(:,:);

ci_x_up = prctile(test,lower_ci_level);

ci_x_down = prctile(test,upper_ci_level);

median_x = prctile(test,50);

% parameter variance

test(:,:) = x_var_stand(:,:);

ci_x_var_up = prctile(test,lower_ci_level);

ci_x_var_down = prctile(test,upper_ci_level);

median_x_var = prctile(test,50);

figure(1); % plot medians and ci's overtop individual lines

subplot(4,6,j); box on;

jbfill(stand_times,ci_prey_up,ci_prey_down,[0.8 0.8 0.8],[0.8 0.8 0.8],1,1); hold on;

h1 = plot(stand_times,median_prey,'-','LineWidth',2,'Color','m');

h2 = plot(t1,y1(:,1),'-k','LineWidth',2);

if j == 1

ylabel('Prey density','FontSize',12);

xlabel('Time','FontSize',12);

end

axis([0 t_max 0 600]);

title(current_param);

subplot(4,6,j+6); box on;

jbfill(stand_times,ci_pred_up,ci_pred_down,[0.8 0.8 0.8],[0.8 0.8 0.8],1,1); hold on;

plot(stand_times,median_pred,'-','LineWidth',2,'Color','m');

plot(t1,y1(:,2),'-k','LineWidth',2);

axis([0 t_max 0 50]);

if j == 1

ylabel('Predator density','FontSize',12);

end

subplot(4,6,j+2*6); box on;

jbfill(stand_times,ci_x_up,ci_x_down,[0.8 0.8 0.8],[0.8 0.8 0.8],1,1); hold on;

plot(stand_times,median_x,'-','LineWidth',2,'Color','m');

h3 = plot([0 t_max],[parameter_to_use parameter_to_use],'-k');

axis([0 t_max 0 2*parameter_to_use]);

if j == 1

ylabel('Parameter mean ','FontSize',12);

end

%legend([h1 h2 h3],'Median GEM solution','ODE solution','Initial value');

subplot(4, 6, j+3*6); box on;

jbfill(stand_times,ci_x_var_up,ci_x_var_down,[0.8 0.8 0.8],[0.8 0.8 0.8],1,1); hold on;

plot(stand_times,median_x_var,'-','LineWidth',2,'Color','m');

plot([0 t_max],[(std_scalar(j)*parameter_to_use)^2 (std_scalar(j)*parameter_to_use)^2],'-k');

if j == 1

ylabel('Parameter variance','FontSize',12);

end

% calculate rates of change in parameter and population size

if j < 5

for z = 1:num_time_steps-1

delta_x(z) = (median_x(z+1) - median_x(z));

delta_n(z) = (median_pred(z+1) - median_pred(z));

end

else

for z = 1:num_time_steps-1

delta_x(z) = (median_x(z+1) - median_x(z));

delta_n(z) = (median_prey(z+1) - median_prey(z));

end

end

current_param

mdl = LinearModel.fit([median_x_var(1:num_time_steps-1); delta_n]',delta_x,'interactions')

figure(3);

subplot(3,2,j);

scatter3(median_x_var(1:num_time_steps-1),delta_n,delta_x, 30, median_x_var(1:num_time_steps-1), 'filled')

xlabel(['Variance of ',current_param]);

ylabel('Population growth');

zlabel('Trait change');

grid on;

figure(4);

subplot(3,2,j);

plot(median_x_var(1:num_time_steps-1),delta_x, 'ok','MarkerFaceColor','k');

title(current_param);

if j == 1

xlabel('Variance','FontSize',12);

ylabel('Trait change','FontSize',12);

end

end

toc

**Code for Figure 5.**

clear; clc; clf;

tic

mass = 1e5; % this is the evolving trait's starting value

r = 0.6; % specify initial ODE parameters

k = 500;

h = 0.005;

d = 0.005;

e = 2.16*mass^-0.5; % allometric function from DeLong et al 2015 AM NAT

a = 0.11e-6*mass^1; % allometric function from DeLong et al 2015 AM NAT

h_2 = 0.75; % define level of heritability

t_max = 200; % time span

prey_init = k; % initial prey density

pred_init = 10; % initial pred density

%% run standard solver on differential Mac Ros equation

y0 = [prey_init pred_init]; % initial prey and predator densities

tspan = [0 t_max]; % start end times

ode = @(t,y) MR_model(t,y,r,k,a,h,e,d); % compile function and call

[t1,y1] = ode45(ode, tspan, y0); % return time and population density vectors

%% Gillespie algorithm

parameters = {'a','e','a&e'}; % group parameters

std_scalar = [0.45 0.45 0.45];

for j = 1:3

current_param = parameters(j);

num_replicates = 200; % number of simulations

stand_times = 0:2:t_max; % standardized time steps for storing time series

num_time_steps = length(stand_times);

n_stand = nan(num_replicates,num_time_steps); % preallocate matrix for standardized population size

p_stand = nan(num_replicates,num_time_steps);

x_stand = nan(num_replicates,num_time_steps);

x_var_stand = nan(num_replicates,num_time_steps);

for i = 1:num_replicates % start Gillespie algorithm

% preallocate for whole time series

n = zeros(1,1e5); % 1e5 is just a large number to ensure the vector is long enough

p = zeros(1,1e5);

t = nan(1,1e5);

x_mean = nan(1,1e5);

x_var = nan(1,1e5);

% define initial states

t(1) = 0; % initial time

n(1) = prey_init; % initial prey population size

p(1) = pred_init; % initial predator population size

% create initial distribution for parameter

rng('shuffle'); % change random number seed

x_dist_init = pearsrnd(mass,std_scalar(j)*mass,0.3,2.5,pred_init,1); % specify initial distribution of traits

while sum(x_dist_init<0) > 0 % if there is a negativein x_dist_init, replace it

x_dist_init(x_dist_init<0) = pearsrnd(mass,std_scalar(j)*mass,0.3,2.5,sum(x_dist_init<0),1)

end

x_dist = x_dist_init; % reset parameter distribution at the start of each simulation

x_mean(1) = mean(x_dist); % initial mean parameter

x_var(1) = var(x_dist); % initial variance in parameter

count = 1; % start counter to index steps while inside loop

while t(count) < t_max

if p(count) > 0 % as long as population size is > 0, pick another individual

R = randi(length(x_dist),1); % randomly choose individual from the vector

x_next = x_dist(R); % pick the parameter for that individual

else x_next = 0;

end

e_next = 2.16*x_next^-0.5; % turn current mass into e

a_next = 0.11e-6*x_next^1; % turn current mass into a

% set up rates of each possible event, given by ODE in MR_model.m

% birth rate of prey

b_n = r*n(count);

% natural death rate of prey

d_n_1 = r*n(count)^2/k; % otherwise r and k are fixed

% mortality rate from predation

if j == 2

d_n_2 = a*n(count)*p(count)/(1+a*h*n(count)); % a is fixed

else

d_n_2 = a_next*n(count)*p(count)/(1+a_next*h*n(count)); % at j = 1 and 3, a evolves

end

% predator birth rate

if j == 1

b_p = e*d_n_2; % e is fixed

else

b_p = e_next*d_n_2; % at j = 2 and 3, e evolves

end

% predator death rate

d_p = d*p(count); % otherwise d is fixed

% sum the events to make wheel of fortune

sum_events = b_n + d_n_1 + d_n_2 + b_p + d_p;

r_num = rand*sum_events; % pick event

% now choose actual events

if (r_num < b_n) % choose birth of prey

n(count+1) = n(count) + 1; % add a prey

p(count+1) = p(count); % hold predator population constant

x_mean(count+1) = x_mean(count); % hold trait mean

x_var(count+1) = x_var(count); % hold trait variance

elseif (r_num >= b_n) && (r_num < b_n + d_n_1) % choose natural death of prey

n(count+1) = n(count) - 1; % take away a prey

p(count+1) = p(count); % hold predator population constant

x_mean(count+1) = x_mean(count); % hold trait mean

x_var(count+1) = x_var(count); % hold trait variance

elseif (r_num >= b_n + d_n_1) && (r_num < b_n + d_n_1 + d_n_2) % choose prey death from predation

n(count+1) = n(count) - 1; % take away a prey

p(count+1) = p(count); % hold predator population constant

x_mean(count+1) = x_mean(count); % hold trait mean

x_var(count+1) = x_var(count); % hold trait variance

elseif (r_num >= b_n + d_n_1 + d_n_2) && (r_num < b_n + d_n_1 + d_n_2 + b_p) % choose predator birth

n(count+1) = n(count); % hold prey population constant

p(count+1) = p(count) + 1; % add an individual to p

off_std = std(x_dist)*(1 - h_2); % specify offspring std given heritability h_2

x_parent = h_2*(x_next - mean(x_dist)) + mean(x_dist);

x_dist(length(x_dist)+1) = pearsrnd(x_parent,0.9*off_std,0.3,2.5,1,1); % assign new individual a trait

while x_dist(end) < 0 % if the trait ends up negative, pick again >> would like to fix this!!!!

x_dist(length(x_dist)) = pearsrnd(x_parent,0.9*off_std,0.3,2.5,1,1);

x_dist(end)

end

x_mean(count+1) = mean(x_dist); % calculate new mean trait

x_var(count+1) = var(x_dist); % calculate new variance trait

elseif (r_num >= b_n + d_n_1 + d_n_2 + b_p) && (r_num <= b_n + d_n_1 + d_n_2 + b_p + d_p) % choose predator death

n(count+1) = n(count); % hold prey population constant

p(count+1) = p(count) - 1; % take away a predator

x_dist = x_dist([1:R-1,R+1:end]); % reduce dist by lost individual

x_mean(count+1) = mean(x_dist); % calculate new mean

x_var(count+1) = var(x_dist); % calculate new variance

x_mean(count+1) = x_mean(count); % hold mean value

x_var(count+1) = x_var(count); % hold variance value

elseif r_num == 0 % added this because when n = p = 0, r_num cannot be less than any rate

n(count+1) = n(count); % keep n the same

p(count+1) = p(count); % keep p the same

x_mean(count+1) = x_mean(count); % hold mean value

x_var(count+1) = x_var(count); % hold variance value

end

t(count+1) = t(count) + exp(-1/sum_events)/sum_events;

count = count+1;

end

% find standardized times and corresponding densities (need for ci's)

for q = 1:num_time_steps

val = stand_times(q); %value to find

tmp = abs(t-val);

[idx idx] = min(tmp); %index of closest value

closest = t(idx); %closest value

n_stand(i,q) = n(idx); % prey at standard time

p_stand(i,q) = p(idx); % pred at standard time

x_stand(i,q) = x_mean(idx); % mean a at standard time

x_var_stand(i,q) = x_var(idx); % var a at standard time

end

end

% calculate ci's for time series

upper_ci_level = 75; % choose ci levels

lower_ci_level = 25; % choose ci levels

% prey abundance

test(:,:) = n_stand(:,:);

ci_prey_up = prctile(test,lower_ci_level);

ci_prey_down = prctile(test,upper_ci_level);

median_prey = prctile(test,50);

% predator abundance

test(:,:) = p_stand(:,:);

ci_pred_up = prctile(test,lower_ci_level);

ci_pred_down = prctile(test,upper_ci_level);

median_pred = prctile(test,50);

% parameter

test(:,:) = x_stand(:,:);

ci_x_up = prctile(test,lower_ci_level);

ci_x_down = prctile(test,upper_ci_level);

median_x = prctile(test,50);

% parameter variance

test(:,:) = x_var_stand(:,:);

ci_x_var_up = prctile(test,lower_ci_level);

ci_x_var_down = prctile(test,upper_ci_level);

median_x_var = prctile(test,50);

figure(1); % plot medians and ci's overtop individual lines

subplot(4,3,j); box on;

jbfill(stand_times,ci_prey_up,ci_prey_down,[0.8 0.8 0.8],[0.8 0.8 0.8],1,1); hold on;

h1 = plot(stand_times,median_prey,'-','LineWidth',2,'Color','m');

h2 = plot(t1,y1(:,1),'-k','LineWidth',2);

if j == 1

ylabel('Prey density','FontSize',12);

xlabel('Time','FontSize',12);

end

axis([0 t_max 0 600]);

title(current_param);

subplot(4,3,j+3); box on;

jbfill(stand_times,ci_pred_up,ci_pred_down,[0.8 0.8 0.8],[0.8 0.8 0.8],1,1); hold on;

plot(stand_times,median_pred,'-','LineWidth',2,'Color','m');

plot(t1,y1(:,2),'-k','LineWidth',2);

if j == 1

ylabel('Predator density','FontSize',12);

end

subplot(4,3,j+2*3); box on;

jbfill(stand_times,ci_x_up,ci_x_down,[0.8 0.8 0.8],[0.8 0.8 0.8],1,1); hold on;

plot(stand_times,median_x,'-','LineWidth',2,'Color','m');

h3 = plot([0 t_max],[mass mass],'-k');

axis([0 t_max 0.5e5 1.5e5]);

if j == 1

ylabel('Trait mean ','FontSize',12);

end

%legend([h1 h2 h3],'Median GEM solution','ODE solution','Initial value');

subplot(4, 3, j+3*3); box on;

jbfill(stand_times,ci_x_var_up,ci_x_var_down,[0.8 0.8 0.8],[0.8 0.8 0.8],1,1); hold on;

plot(stand_times,median_x_var,'-','LineWidth',2,'Color','m');

plot([0 t_max],[(std_scalar(j)*mass)^2 (std_scalar(j)*mass)^2],'-k');

if j == 1

ylabel('Trait variance','FontSize',12);

end

end

toc
